# Experiences of Registered Nurses Providing Care to COVID-19 Patients at Sir Ketumile Masire Teaching Hospital and Sekgoma Memorial Isolation Centre

**DOI:** 10.1177/23779608251338369

**Published:** 2025-05-08

**Authors:** Boitshoko Isaac, Charlene Downing, Nompumelelo Ndlovu

**Affiliations:** 1 Department of Nursing, Faculty of Health Sciences, 61799 University of Johannesburg (UJ), Doornfontein, Johannesburg, South Africa

**Keywords:** Registered nurses, experiences, COVID-19, care, patients

## Abstract

**Introduction:**

The negative effects of COVID-19 on registered nurses (RNs) across the globe are slowly being reported. However, documentation in developing countries like Botswana is limited despite the perilous roles and responsibilities they played in patient care during the pandemic. Understanding the experiences of RNs working in COVID-19 isolation areas offers a body of knowledge that can improve current healthcare systems and assist in better preparing for future public health crises.

**Aim:**

To explore and describe the experiences of RNs who worked in selected COVID-19 isolation centres in Botswana.

**Methods:**

An exploratory, descriptive, phenomenological research design was used. RNs were purposefully sampled, and data were collected using phenomenological, in-depth, individual interviews. Giorgi's method of qualitative data analysis was used to analyse the data.

**Results:**

Nineteen RNs aged 23–47 years were interviewed. Three themes emerged with subthemes: participants expressed professional challenges in providing care and treatment, participants made personal sacrifices and felt isolated, and participants shared positive experiences (silver lining) of providing care during the COVID-19 era.

**Conclusion:**

The participants experienced both personal and system challenges but grew professionally and demonstrated resilience. The results pointed to a need for RNs to be supported when providing care during pandemics primarily to enhance their well-being.

## Background

The COVID-19 pandemic has posed unprecedented challenges to Health Care Systems (HCS) globally, with Botswana not being an exception. Registered nurses (RNs), as the backbone of most HCSs, faced significant burdens in adapting to rapidly changing circumstances while providing care for COVID-19 patients. Their pandemic experience has been characterised by physical, emotional, and psychological challenges (Villar et al., 2021; Wu et al., 2024; Zamanzadeh et al., 2021). However, documentation of RNs experiences in developing countries such as Botswana is largely lacking.

The World Health Organization (WHO) declared COVID-19 a pandemic, in January 2020 (Botswana COVID-19 Presidential Taskforce, 2020). During the pandemic, RNs faced unprecedented challenges, resulting in significant physical exhaustion and mental health pressures (Taheri-Ezbarami et al., 2023; Villar et al., 2021; Wu et al., 2024; Zamanzadeh et al., 2021). Although the above authors studied RNs’ experiences, they did so in contexts, cultures, and HCS differing greatly from Botswana's, except for one done in Gaborone by Kealeboga et al. (2023) which also recommended further research on the topic to broaden the understanding of nurses’ experiences nationwide.

Botswana confirmed its first COVID-19 case on March 20, 2020 (Chatibura, 2020; Motlhatlhedi et al., 2020). The country immediately, implemented the WHO's COVID-19 Strategic Preparedness and Response Plan (SPRP) to shape its Country Preparedness and Response Plan (CPRP) (Masiya et al., 2021). This led to the formation of eleven isolation centres, adding to the existing 26 public hospitals for COVID-19 services (Olashore et al., 2022). The CPRP focused on four strategic measures: active surveillance, early detection, case management, and contact tracing. RNs played a crucial role in executing these strategies, (Siamisang et al., 2022). While the situation appears to have stabilised, the emergence of new variants still poses a threat. The country continues to prioritise the provision of essential health services with less attention on frontline service providers, RNs. Given that RNs formed the majority of Botswana's HCS health workforce during the pandemic, they are likely to have faced a greater negative pandemic impact compared to other healthcare workers. Even in the realization of such unpalatable occupational threats, little has been done to understand the experiences of RNs in Botswana. Therefore the main aim of this study was to explore and describe the experiences of RNs who worked in selected COVID-19 isolation centers in Botswana.

## Methods

### Study Design

A qualitative, exploratory, descriptive research design with a phenomenological approach was utilised in this study. This hybrid approach is recommended by Hunter et al. (2019) and Polit and Beck (2021) when the topic under investigation has received limited previous attention and can provide a deep, nuanced understanding of participants’ experiences, thoughts, and feelings.

### Study Setting

The study was conducted at two of Botswana's District Health Management Teams (DHMT): Greater Gaborone (Sir Ketumile Masire Teaching Hospital) and Greater Palapye (Sekgoma Memorial Isolation Centre). [Sir Ketumile Masire Teaching Hospital] is a teaching hospital with a 450-bed capacity which according to Siamisang et al. (2022) was used as the country's only COVID-19 public high-care and referral hospital. Sekgoma Memorial Isolation Centre, is located in one of Botswana's largest villages with approximately 118 beds capacity.

### Population and Sampling

The study population consisted of RNs assigned to the two isolation centres to provide care for COVID-19 patients. Purposive sampling was used to recruit participants who met the inclusion criteria. The approach ensures the representation of relevant variations based on existing literature, prior experience, and theoretical considerations. (Busetto et al., 2020).

#### Inclusion Criteria

All RNs admitted to the Nursing and Midwifery Council of Botswana (NMCB) with over year experience. RNs were expected to have practised for more than three months at the COVID-19 isolation centres. Data were generated from nineteen (19) participants purposively sampled from both study sites (twelve from Sir Ketumile Masire Teaching Hospital [SKMTH]) and seven from (Sekgoma Memorial Isolation Centre (SMIC).

### Recruitment Procedure

The researcher gained access to both research sites through the hospital managers, who served as gatekeepers. After receiving the necessary study information and permission letters, the managers distributed this package to the nursing in-charge officers and unit managers, who then shared it with their staff. Subsequently, the researcher was invited to visit various units, including Internal Medicine, Accident and Emergency, Radiology, and the Staff Clinic at SKMTH, as well as Accident and Emergency, ICU, Postnatal Ward, and Gynaecology at SMIC. During these visits, the researcher provided potential participants with comprehensive information about the study and encouraged their participation. Those interested were invited to schedule appointments with the researcher. Before obtaining consent, the researcher clearly communicated all potential risks and benefits, which were also included in the study information sheet, forming the first part of the consent form. This allowed participants to make informed decisions about their involvement. All participants who met the inclusion criteria agreed to participate, providing written consent and permission to be audio-recorded.

### Data Collection

The interviews were piloted with four participants at SKMTH to evaluate the interview guide and help the researcher to familiarise herself with the data collection process. Data collection took place from April 24 to November 30, 2023 which was towards the end of COVID-19, at that time the two isolation centres had no patients. During the data collection period, the researcher travelled between SKMTH and SMIC. The researcher triangulated several data collection techniques, which were individual in-depth face-to-face phenomenological interviews and field notes. According to Polit and Beck (2021) and Stahl and King (2020), using multiple methods can enhance credibility in qualitative research.

The interviews were conducted in a comfortable environment where participants felt safe to share their experiences without distractions and at a time convenient for them. Suitable locations included meeting rooms, nurse in charge's office, unused nurses’ stations, and participants’ homes in the garden. The interviews were conducted with lead author and RNs only and used one guiding question that was designed to inquire into participants’ lived experiences of the phenomenon, “*How was it for you to provide nursing care to COVID-19 patients at SKMTH/SMIC.* The researcher followed up with probes based on participants’ responses, as described by Alirezaei and Roudsari (2020). The interviews lasted for an average of 52 min 38 s with shortest interview at 27minutes 55 s and the longest 1 h 22seconds. The researcher having served during the HIV/AIDS pandemic ran the risk of being biased however bracketing was deployed to ensure that only the voices of the participants were heard.

The observational field notes were recorded shortly after the interviews to capture occurrences that may be missed by an audio recorder, like environmental contexts, behaviours, and nonverbal cues. Personal field notes reflected the researcher's emotions which ranged from comfortable, confident, anxious or excited reflection. While methodological field notes were concerned with researcher's reflection on data collection techniques which included limiting interviews to an hour to prevent participant fatigue and using two recording devices for reliability. Theoretical field notes were recorded to reflect on the interaction between researcher and participants. For example, the researcher found engaging with participants to be generally easy but noted the need to probe further on less-discussed topics. In another instance, participants were open about their experiences, allowing for smooth follow-up questions that were adequately addressed.

Data collection continued until no new insights emerged, which started to be noticeable at Participant 14. However, five additional interviews were conducted to ensure adequate representation of RNs from different units like ICU, Maternity and ER as well as to probe emerging themes further for a richer, more nuanced understanding.

### Data Analysis

Data analysis occurred concurrently with data collection, utilizing Giorgi's reductive phenomenological psychological method, which according to Giorgi et al. (2017a,b) involves a five-phase process. Phase 1 of getting to know the data, the researcher repeatedly listened to all 19 interview recordings of participants sharing their experiences, these were then transcribed and read several times to get a holistic view of what participants were conveying and their tone. On the second phase of identifying meaning units, the researchers went through the transcripts and read line by line to identify meaning units (a general feel of what the participants was conveying in that data segment). Over 400 meaning units were extracted and reported as either single words like “ hectic/draining/hopelessness” or full statements like “ I was afraid of bringing the virus back home”. The researchers meticulously noted all paralinguistic communications from transcriptions and theoretical notes, e*xamples included participants rubbing their faces or hands, tapping pen, swinging chairs uncontrollably, shaky or low tone voices, teary eyes.*

The third phase was regrouping unit meanings into clusters, the researchers transformed each meaning and reworded it into a phenomenological expression that can be linked to the research question. For example “ hectic/draining/hopelessness” “*shaky or low tone voices, teary eyes*” became “Physical, Emotional strain and dysregulation accompanied by compassion fatigue”. “I was afraid of bringing the virus back home” became “excessive occupational viral exposure & fear of infecting family members”. On the fourth phase of identifying the essential structure of the phenomenon, the researchers organised and transformed unit meanings based on their commonalities and relations to come up with central themes and subthemes. The last stage of integrating features into the essential structure of the phenomenon saw the researchers reaching out to some of the participants to confirm if the developed themes and subthemes captured their experience. Also the research team held three meetings (18.01.24 9am to 11am, 25.01.24, 9am to 11am and 15.02.24, 1pm to 2pm) to discuss the proposed themes, integrate, and modify classification and categorization into the themes and subthemes. Raw data and confidentiality form were handed to the independent coder on the 21st of February 2024. The lead researcher and independent coder meet and agreed on the final codes and themes at a virtual meeting on the 29^th^ February 2024 from 7pm to 9.30pm. The rest of the research team received the report from the independent coder (06.03.24 12-1230) and agreed on the final psychological structure of the RNs experience as presented in Table 1.

**Table 1.** Participants Demographic Data.

Code	Age	Gender	Experience as a Nurse (Years)	Experience in REDACTED (Months)
L1	23	M	2	18
L2	32	F	19	28
L3	40	F	17	18
L4	35	M	14	20
L5	39	F	17	30
L6	37	M	15	15
L7	37	M	14	24
L8	47	M	25	23
L9	27	M	5	19
L10	26	F	3	15
L11	32	M	11	27
L12	38	M	17	24
L13	35	M	12	24
L14	47	M	21	11
L15	36	F	16	6
L16	36	F	14	6
L17	37	M	16	14
L18	27	M	4	16
L19	38	F	13	11

### Measures to Ensure Trustworthiness

Cypress (2017) and Polit and Beck (2021) define trustworthiness as the quality, authenticity, and truthfulness of qualitative research findings. To establish trustworthiness, the researchers adhered to four criteria: credibility, transferability, dependability, and confirmability (Cypress, 2017; Polit & Beck, 2021; Stahl & King, 2020). Strategies to enhance credibility, included prolonged engagement, persistent observation, researcher triangulation, and member checking. Data collection triangulation was achieved through field notes and one-on-one interviews. An independent coder was involved to minimize investigator bias, and findings were reported using participants’ original quotes. Transferability is analogous to the generalisability of study findings to different settings, researchers ensured this by choosing different settings rural and standard care (SMIC) and urban high care (SKMTH). Moreover, a dense description of the participants, research environments, processes and findings was offered. The research process was thoroughly documented to allow for replication, ensuring the stability of the data over time.

### Ethical Considerations

The researcher obtained permission to conduct the study from the Faculty of Health Science Research Ethics Committee and Higher Degrees Committee in University of Johannesburg, South Africa and the University of Botswana Institutional Review Board Institutional Review Board. The researchers also sought ethical clearance from Botswana Ministry of Health, Research, Science & Technology, Greater Palapye DHMT and SMIC and SKMTH IRB committees. Moreover, the ethical principles of informed consent, confidentiality, justice, scientific integrity, as well as a thorough explanation of risks and benefits to participants were applied to ensure participants protection.

## Findings

### Participants Demographic Data

Nineteen participants, (12 males, 7 females) aged 23 to 47, were interviewed. All held a diploma or degree in nursing and were registered with the NMCB as RNs. They had 2–25 years of working experience as RNs and had served in a COVID-19 isolation centre for 6 to 30 months. Table 1 presents the demographics of the participants.

### Central Storyline

The researchers found that the RNs’ experiences commenced with confusion and uncertainty due to HCS's unpreparedness and lack of familiarity with pandemic response. This resulted in a hectic work environment, fear of COVID-19 infection, social stigma, and isolation. Evenso, the pandemic presented opportunities for nursing care thoroughness and accuracy, enabling nurses to advance their professional practices, both psychologically and spiritually.

### Themes and Subthemes

The themes, together with the eleven identified subthemes, are presented below in Table 2.

**Table 2.** Summary of Themes and Subthemes.

THEME	SUBTHEME
Theme 1: Participants expressed professional challenges in providing care and treatment.	a) Lack of knowledge, confusion and uncertainty about the pandemic b) Hectic work environment and providing nursing care in a low-resourced setting c) Emotional strain and dysregulation accompanied by compassion fatigue d) Inconsistent and ineffective managerial support
Theme 2: Participants made personal sacrifices and felt isolated.	a) Excessive occupational exposure and fear of infection with COVID-19 b) Separation, isolation and stigma c) Dysfunctional psychological regulation and coping
Theme 3: Participants shared positive experiences (silver lining) in providing care during the COVID-19 era.	a) Good teamwork and interpersonal relations b) Pleasant social experience and inner satisfaction c) Career and personal growth d) Faith-based growth

#### Challenges in Providing Care and Treatment

Narrations from the participants critically synthesised evidence of confusion and uncertainty due to a lack of system preparedness, sufficient knowledge, training and education on how to manage the pandemic. The absence of prior experience with disaster or pandemic management, coupled with the perplexing trajectory of the disease's spread, created a pervasive sense of ambiguity as shared by Participant 5:

“*… we did not know the management, it differed and kept changing time and again, and everybody was trying their way with nothing to guide their decision. You will see that today we do this and next thing we have changed, mainly because those ways would have not resulted in good outcome”* L5, F/39 years.

Participant 12 shared that the experience of not knowing was expressed in the metaphor of that of a soldier taken to war, highlighting uncertainty.

“*It was like being soldiers taken to war, anything can happen in the battlefield”* L12, M/38 years.

Participants from both study sites expressed difficulties of working in COVID-19 isolation centres mainly because of their workload. One participant shared:

“*My first shift was night duty iyoooh! iyoooh! That night, I was on my feet the whole shift, literally, I never sat down, the whole night. We had critically ill patients with high-end respiratory care needs, and there was only three us nursing over 40 patients. It was always busy; I remember that in the four days that you will be working, you won’t touch your phone, there was no time”* L6, F/37 years.

Participant L11 shared how he had to work more than 12 h, and it was very common for these shifts to stretch beyond the set 12 h due to patient care demands.

“*There was so much pressure, that 12-h shift was not a joke, sometimes we will knock off at 11pm at night since 7am in the morning. We would start giving report to the incoming shift nurses and as you are doing that more patients are coming in you have to attend to them, stabilize them and go back to the report”* L11, M/32 years.

The pandemic brought into sharp relief the gap between resource supply and demand, which already existed before COVID-19, reporting shortages of beds, working space, and oxygen. There was also a shortage of human resources, and teams were composed of newly trained nurses and doctors. Participant 14 shared

“*…. you will see that other hospitals were fully equipped; they had blood gas analysers, enough sip-up masks, had a fully functional ICU with constant oxygen supply, equipment to monitor a coagulation profile, and effective radiology departments. These things were lacking in our set-up, the ward should have been run by a specialised doctor, not a junior general practitioner”* L14, M/47 years.

In both facilities, it became clear that other hospital departments did not have staff assigned to COVID-19, compromising the quality of care as reported by L14.

“*It was a stressful environment with other departments not fully participating in patient care. Sometimes we will be told that our patients will be attended to after hours, then the doctor will only come to review the X-ray the following day and the patient dies that very same night. We had patients who had respiratory problems and we will refer them to a respiratory physiotherapist, and they won’t show up. We had some of our patients with chronic medical conditions like diabetes mellitus who needed dieticians’ review, but they won’t get help; other departments like radiology, dietetics, physiology were not really supportive. It was like we owned these patients, alone for that matter”* L14, M/47 years.

The COVID-19 era was described as an emotionally challenging period causing emotional strain and dysregulation accompanied by compassion fatigue. Participants in our study reported experiencing a high death rate, leaving them devastated, traumatised and numb.

One participant shared their experience:

“*Iyoooh! It was heavy, emotionally heavy! Sometimes you will feel so helpless, you will hide in the bathroom and cry it out, cry alone, wipe those tears and come back to the ward to continue working; there won’t be anyone to ask “What is the problem?” “Are you okay?”. When you get back to the ward you find that patients have died and you have to do last office preparations the whole shift, it was not easy; we didn’t have time to catch some breath, let alone breathe”* L5, F/39 years.

All these repeated experiences of loss and suffering triggered moments of emotional withdrawal and compassion fatigue, as drawn from Participant 12's experience.

“*Most of us endured the experience of seeing another person going through pain; initially, I had moments where I would lock myself in the bathroom and let my tears roll when I lost a patient (laughs), and I later on figured I will get myself sick, we have to move on”* L12, M/38 years.

Participants gave an account of varying managerial support, with some saying they felt supported, while some said the support came late or was never there. For example, Participant 5 shared that:

“*Towards the end of the project, that's when we were offered counselling, but it was already late, and I found it useless because we had given into the struggle and had somehow toughened up. So, ah!”* L5, F/39 years.

While participant 18 said:

“*I can’t talk of any support that I can say was helpful. What I know is that by the time we left the isolation centre, it was almost like we had gone there to avoid regular hospital work or to be idle”* L18, M/27 years.

#### Participants Made Personal Sacrifices and Felt Isolated

Participants shared facing high occupational risk of being infected with COVID-19 virus owing to prolonged pathogen exposure in a hectic environment prone to infection control errors and sometimes inadequate PPE protection. Their account of the experience was filled with descriptions of how they lived with constant fear of getting infected or transmitting the infection to their families, colleagues and friends. Participant 5 shared that:

“*I could not stay here because I had a small baby, so I always took the risk to go home; it was very risky. It was scary; you will never know if you did enough disinfection because the main aim was to protect the baby and the entire family so it was scary”* L5, F/39 years.

Owing to their fear of infecting their significant others, they had to isolate to reduce interactions with them, resulting in strong feelings of social separation and isolation. Participant 12's experience in this regard echoed the separation:

“*We were isolated from our families and one thing that was awkward was being separated from our patients for infection control; we were forced to keep our distance, so our patients were admitted in this cubicles with clear, transparent glasses; we even switched off door motion sensors so that we keep the doors closed”* L12, M/38 years.

Participants reported feelings of social rejection and stigma both at work and in public, expressing concerns about being perceived as “dirty” due to their workplace. They were surprised to experience stigma from colleagues, believing their peers were well-informed about their roles.

Participant 19 mentioned that:

“*Nobody came to us, we were not allowed to go to any part of the hospital, management did not come near the ward, there was so much stigma. Nobody wanted to interact with us; it was as if we were from a different planet”* L19, F/38 years.

They shared that communities used words and statements that demonstrated stigma. They were considered COVID-19 carriers, and nobody wanted to see them.

“*….even in the community, the moment they learn that you work in a COVID centre, they want you to stay as far possible as you can from them. At the shops or service centres, the moment they identify you they give space to pass and be assisted so you can go, a part of me felt that it was stigma. Because some will even verbalise it, saying, “hey please assist before she infect us”* L3, F/40 years.

At some point, participants came to a place where they felt they were “running on empty”, sharing the inability to cope or over-relying on unhealthy coping strategies. These maladaptive strategies, included avoidance tactics, as well as use of alcohol and substances. Participant L1 said:

“…O*h, those are the emotions that I really had to keep suppressed because if I ever did, it would make me feel some type of way, and I do not like feeling that way”* L1, M/23 years.

Some participants mentioned that most of them started using alcohol and substances to survive this painful experience.

“*You know, during Covid, most of us succumbed to alcohol usage; when I came here, I wasn’t drinking alcohol (laughing); I started here. I started drinking in 2021, it was a way of postponing the stress or dealing with issues as hand, you see”* L7, M37years.

Some of these behaviours remain with them to date.

“*I even started bad habits since that time, things that I never used to do, so yeah, I started smoking marijuana. I almost lost my wife because of it; she had a problem with the fact that I was smoking. Now I'm a smoker and it's difficult to quit”* L17/M37 years.

#### Participants Shared Positive Experiences (Silver Lining) While Providing Care During COVID-19

Participants shared positive experiences, emphasizing good teamwork, personal and professional growth, strengthening of their faith and resilience. Their faces lightened when sharing this experience, noting it as the best team spirit they had experienced in their careers. They described unparalleled support among nurses, united in the common goal of making their work easier through team spirit.

“*There was good teamwork. Heish! A lot was at stake; we did not have any other choice but to pull through together, and we did it. Everybody was in it to win. So we worked and supported each other very well”* L19, F/36 years.

The COVID-19 pandemic was also reported to have been a pleasant social experience and brought inner satisfaction, especially during positive health outcomes. RNs reported enjoying interactions with patients from all walks of life, including people of high social standing. They also shared experiences of feeling a good sense of inner satisfaction when they fulfilled patient's needs. Participant 15 shared that:

“… so by the time I knocked off, I will be so proud that I did something. Even those small things like feeding a patient who could not eat, I was proud of such moments” L15, F/36 years.

RNs managed to seize the moment and exploited this extraordinary chance for personal and professional growth. Participants shared that the pandemic presented an opportunity for them to learn new innovations in nursing practice, such as the use of new technologies for patient monitoring, telemedicine, and online education. They grew in confidence to deliver high-quality nursing care, preventing complications and treating patients confidently and successfully to recover. Participant 18 shared that:

“*When I volunteered, it was a new disease, and one thing I knew was it was a learning opportunity. It was new then, and so we were sort of a vanguard in terms of available knowledge. I also am someone who likes to venture into new areas and explore, so it gave me that opportunity”* L18, M/27years.

Indeed, learning did take place; they learnt a lot through COVID-19.

“*So we had to learn how to detect and diagnose conditions earlier. I remember we have to come up with this strategy that every patient who came here, we have to routinely screen them to RBS and check ketones because most of our patients would be diabetic or, even if somebody was prediabetic, COVID will swing them to diabetes fast before you know it is now DKA so it kept us on our toes, we had to read and try and see how other countries are doing it and also to try and be competent so that we can handle the pressure”* L11/M32 years.

Given the challenges that came with the COVID-19 pandemic, participants shared that they fought the virus with everything, including relying on a supreme power. They reported that patients were also allowed to worship their gods through prayer and music. They drew strength from this belief that a supreme power was with them always. Participant L10 reported:

“*We would talk and pray, most of the time it was prayer, every day when we get to work we would pray, later on during the day when something tragic like death happens, we would come together and pray again, so our supervisor believed so much in prayer and she was there for us”* L10, F/26 years.

Nurses also had a chance to experience and practise transcultural nursing; they said this was a result of a new development of allowing cultural practices in the hospital.

“*… the family came here to perform rituals to collect his spirit, and they were allowed. They believed that his spirit is here and they needed to carry it home, it's interesting how our healthcare system was so flexible and permissive during COVID, it was something we have never seen, and they were allowed which I feel was good”* L12, M/38 years.

## Discussion

This study aimed to describe the experiences of RNs providing care to COVID-19 patients at two isolation centres in Botswana. The findings reveal significant systemic and personal challenges like lack of preparedness, overwhelming workloads, emotional strain, stigma, and social isolation. Similar findings were reported in studies by Suplee et al. (2022), Crowley et al. (2021), Khademi and Imani (2022), and Lee et al. (2023), noting that governments employed hurried measures to tackle the crisis, subsequently pushing RNs into the battlefield without sufficient training. Additionally, conflicting and rapidly changing clinical guidelines contributed to confusion and frustration among RNs, a situation which mirrored in Botswana as indicated by the current study, as well as findings from Kealeboga et al. (2023) and Seloilwe et al. (2023)

In contrast, the findings of Al-Ashwal et al. (2020), Han et al. (2023), and Al Awamleh (2021) differed. Most nurses in these studies reported feeling knowledgeable and confident in caring for COVID-19 patients, attributing this to adequate training and well-equipped institutions with effective disaster management, infection control, and treatment plans. These factors contributed to the nurses’ preparedness in COVID-19 service areas, whereas the current study highlighted a lack of such resources in both isolation centres.

Numerous studies Lee et al. (2023), Lavoie-Tremblay et al. (2022), Jarrar et al. (2023), and Martin et al. (2023), reported high nurse-patient ratios, extremely long working hours, and sacrificed rest days due to staffing shortages during the pandemic coupled with resource shortages. Lucchini et al. (2020) and González-Gil et al. (2021) noted a significant imbalance between workload and available human resources, with Lucchini et al. (2020) reporting a 33% increase in workload due to the high demand for critical care, including mechanical ventilation and PPE usage. Consistent with findings of the above studies, the current study revealed shortages of nurses, inexperienced newly trained staff, acute care beds, and essential supplies like oxygen and PPE. Additionally, the study found that the usual multi-professional team (MPT) collaboration that existed before COVID-19 was largely absent in Botswana during the pandemic. This contrasts with studies by Rogers et al. (2023) and Barber et al. (2023), which praised MPT efforts in various specialties, likely attributed to training that emphasized the significance of collaborative MPT in pandemic response, which lacked in the in the current study.

Nurses’ experiences during the COVID-19 era were described as surreal and unreal in studies by Alwesmi et al. (2022), Castaldo et al. (2022), and Gómez-Brufal-Flores et al. (2024). These studies reported alarming death rates, with Pilbeam and Snow (2022) noting that even in ICUs, COVID-19 deaths were shocking, often involving patients who seemed to have a better chance of survival. Participants in these studies, like those in the current research, shared experiences of witnessing numerous deaths, describing COVID-19 deaths as profoundly different from other types. Sperling (2024) and Alwesmi et al. (2022) revealed the emotions associated with this trauma, including shock, sorrow, guilt, disbelief, frustration, and emotional burden. Jarrad et al. (2024) noted that the COVID-19 pandemic was marked by adversity and hardship, leading some nurses to adopt coping strategies to escape reality like harmful alcohol and substance use, such as painkillers, anti-anxiety drugs, coffee, and cigarettes. Jarrad et al. (2024) and Kowalczuk et al. (2022) also reported the use of avoidance techniques among nurses. Despite differences in geography, methodology, and sample sizes, these studies reached similar conclusions, aligning with the findings of the current study.

Support between managers and nurses carries a history, however, the pandemic seemed to have highlighted its importance even more, with nurses reporting various types of managerial support. Skogsberg et al. (2022) found that the majority of the participants reported good managerial support, whereas some reported that they did not. This finding was consistent with studies performed in Saudi Arabia, South Africa and Botswana by Nasaif et al. (2023), Dikobe et al. (2023), Molala and Downing (2023) and Kealeboga et al. (2023) respectively. In contrast, findings from Dobnik and Mateja (2023) and Xie et al. (2023) indicated that nurses did not report feelings of being unsupported by their managers. Participants in these studies expressed high levels of job satisfaction. They reported receiving adequate information, efficient teamwork, sufficient personal safety equipment, and appropriate infection protection, leading to a sense of job security by the end of the pandemic.

Numerous studies, including those by Labrague and de Los Santos (2021), Moradi and Sharififar (2022), Dikobe et al. (2023), Alhalaiqa et al. (2023); Halcomb et al., 2020), Vejdani et al. (2021) and Kealeboga et al. (2023), have reported nurses’ fear of COVID-19 infection just like in the current study. In this study, this fear was most pronounced at the beginning of the pandemic but appeared to lessen as nurses became more accustomed to their roles and accepted the risks involved. In contrast, Koiwa et al. (2022) and Chung et al. (2022) found that nurses in Japan, Hong Kong and Taiwan had no fear of contracting COVID-19. These regions had prior experiences with pandemics like SARS and MERS, which likely improved their pandemic preparedness and infection control measures, reducing fear.

Popoola et al. (2022) reported that nurses believed providing care to COVID-19 patients would enhance their careers and knowledge. Similarly, Chau et al. (2021) and Dikobe et al. (2023) noted that nurses became more knowledgeable, capable, adaptive, and confident in delivering life-saving care. These findings resonate with the current study, where nurses reported gaining skills in using new equipment and managing complications such as hypertension and diabetes.

The findings of this study provide the core themes of emotional resilience and importance of timely response resource and support wise which can transferred to high-stress work environments like disaster preparedness teams, ICU and ER. These major themes can be transferred to manage future pandemics of this nature. For example the study revealed preexisting HCS issues such as lack of resources, health care training and mentorship programs, inadequate health care infrastructure which is crucial for building more resilient work force and HCS. The implication of these findings for policy and practice are significate and can be transferred to inform strategies to promote resilience. Future research should develop and evaluate programs to enhance the well being of RNs during pandemics or high-pressure areas.

## Limitations

The researcher recognised the possibility of some participants not revealing all their experiences due to worries about possible consequences and some choosing to suppress their feelings or avoid remembering some parts of the experience. However, an effort was made to handle this limitation as much as possible by assuring the participants of the confidentiality and anonymity of their information. Also, participants were offered a safe space to share their experiences as well as counselling services where necessary.

## Implications for Practice

This study documented RNs’ experiences during the COVID-19 pandemic in Botswana and revealed critical insights that can inform nursing practice and healthcare policies. These include enhanced preparedness through training and drills focused on infectious diseases emergency response as well as crisis management to reduce confusion in future pandemics. Moreover, health care institutions should invest and prioritise provision of supportive work environment like safe work practices and workload management, strong team building and mental health resources to enhance the well-being of RNs during pandemics like COVID-19.

## Conclusion

The experiences of RNs while providing care to COVID-19 patients in REDACTED have accentuated the vital role they play in the healthcare system, and highlighted their resilience in the face of adversity. However, these experiences also illuminate the pressing need for improved working conditions, adequate emotional support, and structured governmental strategies to ensure that nurses can effectively cope with the challenges of future healthcare crises.

## Supplemental Material

sj-pdf-2-son-10.1177_23779608251338369 - Supplemental material for Experiences of Registered Nurses Providing Care to COVID-19 Patients at Sir Ketumile Masire Teaching Hospital and Sekgoma Memorial Isolation Centre Supplemental material, sj-pdf-2-son-10.1177_23779608251338369 for Experiences of Registered Nurses Providing Care to COVID-19 Patients at Sir Ketumile Masire Teaching Hospital and Sekgoma Memorial Isolation Centre by Boitshoko Isaac, Charlene Downing and Nompumelelo Ndlovu in SAGE Open Nursing

sj-pdf-3-son-10.1177_23779608251338369 - Supplemental material for Experiences of Registered Nurses Providing Care to COVID-19 Patients at Sir Ketumile Masire Teaching Hospital and Sekgoma Memorial Isolation Centre Supplemental material, sj-pdf-3-son-10.1177_23779608251338369 for Experiences of Registered Nurses Providing Care to COVID-19 Patients at Sir Ketumile Masire Teaching Hospital and Sekgoma Memorial Isolation Centre by Boitshoko Isaac, Charlene Downing and Nompumelelo Ndlovu in SAGE Open Nursing
