# Supplementary material for: Experiences of Registered Nurses Providing Care to COVID-19 Patients at Sir Ketumile Masire Teaching Hospital and Sekgoma Memorial Isolation Centre
Source: SAGE Open Nurs. 2025 May 8;11:23779608251338369. doi: 10.1177/23779608251338369 (PMC12064891; doi:10.1177/23779608251338369)
Supplement: sj-pdf-3-son-10.1177_23779608251338369 - Supplemental material for Experiences of Registered Nurses Providing Care to COVID-19 Patients at Sir Ketumile Masire Teaching Hospital and Sekgoma Memorial Isolation Centre [file sj-pdf-3-son-10.1177_23779608251338369.pdf]

**Interview Guide**

The main interview questions posed to the participants will be as follows:

1. How was it for you to provide nursing care to COVID-19 patients at SKMTH/SMIC

Follow-up questions to be posed to participants will be:

1. What are the main psychological feelings you experience while nursing Covid-19 patients?
2. How do you cope with changes in your work and life?
3. How did you feel when accepting these new roles?
4. What were your expectations when accepting this new role?
5. What are your thoughts and feelings about this new role?
6. What kind of support if any, do you receive from your institution to enhance your well-being while executing this role?
